# A Case of Psychogenic Myoclonus Responding to a Novel Transcranial Magnetic Stimulation Approach: Rationale, Feasibility, and Possible Neurophysiological Basis

**DOI:** 10.3389/fnhum.2020.00292

**Published:** 2020-07-17

**Authors:** Antonino Naro, Loris Pignolo, Luana Billeri, Bruno Porcari, Simona Portaro, Paolo Tonin, Rocco Salvatore Calabrò

**Affiliations:** ^1^IRCCS Centro Neurolesi Bonino Pulejo, Messina, Italy; ^2^Sant’Anna Institute, Research in Advanced Neurorehabilitation (RAN), Crotone, Italy

**Keywords:** functional connectivity, premotor cortex (PMC), psychogenic movement disorders (PMDs), psychogenic myoclonus, repetitive transcranial magnetic stimulation (rTMS)

## Abstract

Repetitive transcranial magnetic stimulation (rTMS) can relieve motor symptoms related to psychogenic movement disorders (PMDs), but the subtending neurophysiological basis is unclear. We report on a 50-year-old woman with a diagnosis of psychogenic myoclonus in the right lower limb, who was treated with a daily session (in the late morning/early afternoon) of 1 Hz rTMS over the left premotor cortex (PMC), five times a week for 6 weeks. Clinical data and EEG at rest were collected before and immediately and 2-month after the rTMS protocol completion. The patient reported a significant reduction of involuntary movement frequency and intensity and the related disability burden up to the follow-up. In parallel, any abnormality in terms of source current density within and connectivity between the frontal and parietal areas was reset. The short follow–up period, the lack of extensive neurophysiological measures, and the lack of control treatment represent the main limitation of the study. However, low-frequency rTMS over PMC seems a safe and promising approach for the management of psychogenic myoclonus owing to the combination of cortical neuromodulation and non-specific mechanisms suggesting cognitive-behavioral effects.

## Introduction

Movement disorders (MDs) are clinical syndromes characterized by either involuntary movements (hyperkinetic MD) or a paucity of movements (hypokinetic MD; Fahn et al., 2011; Donaldson et al., 2012; Morgante et al., 2013; Martino et al., 2016). MD can be either organic (i.e., idiopathic or secondary to a systemic or neurologic disorder) or psychogenic (PMD), that is, they are not attributable to any structural or neurochemical pathology. PMDs represent the latent symptoms of psychiatric illness or simulation, including motor subtype of conversion disorders (Dallocchio et al., 2015; Hallett, 2016; Barbey and Aybek, 2017). Organic MDs and PMDs share the same symptoms substantially. However, motor symptoms in PMDs are often complex (e.g., two or more symptoms), involve multiple body parts, are variable in time and bodily distribution, and sensible to placebo administration or patient’s clinical observation (Thenganatt and Jankovic, 2019). Furthermore, PMD is characterized by the inconsistency/incongruency, abrupt onset, distractibility, and oddness of the involuntary movements (Fahn and Williams, 1988; Williams et al., 1995; Peckham and Hallett, 2009; Hallett, 2016). PMD diagnosis remains difficult for both the neurologist and the psychiatrist. An electrophysiological examination is a useful tool for evaluating and supporting the diagnosis of PMD. It includes accelerometry, surface electromyography, electroencephalography (EEG), somatosensory evoked potentials, and transcranial magnetic stimulation (TMS; Peckham and Hallett, 2009). Also, PMD pathophysiology is still not understood completely. In particular, abnormal connectivity between the limbic and motor networks, altered top-down regulation of motor activities from the anterior cingulate cortex (ACC) and insular cortex, and a decreased activation of the supplementary motor area (SMA) and pre-SMA are among the main pathophysiological features of PMDs (Baizabal-Carvallo et al., 2019). However, organic and PMD share some neurophysiological features including decreased cortical inhibition and abnormal thalamocortical connectivity (Baizabal-Carvallo et al., 2019).

Appropriate management of PMDs is essential, as these lead easily to disability and suffering, and become chronic if untreated (Jankovic and Sherer, 2014; De Keersmaecker et al., 2019). Physical, speech and occupational therapy are reported as useful to improve patient’s functioning and are proposed to reprogram the abnormal movement pattern (namely, motor reprogramming). Furthermore, antidepressants and muscle relaxants may also be beneficial (Espay et al., 2009; Gelauff et al., 2014; Ricciardi and Edwards, 2014). When these approaches are unsuccessful or not tolerated by the patient, non-invasive brain stimulation has been proposed as a useful add-on to improve a person’s functioning (Nicholson and Voon, 2016; Naro et al., 2019a,b). Specifically, a few studies proposed repetitive TMS (rTMS) as a clinically valuable tool to improve PMDs. The rationale of employing rTMS in PMD is 2-fold, as the therapeutic benefit of rTMS could be due to either a cognitive-behavioral or a cortical neuromodulation effect or both (Pollak et al., 2014; Nicholson and Voon, 2016; Garcin et al., 2017; Taib et al., 2019). However, the underlying mechanisms deserve further investigation (Nicholson and Voon, 2016). Most of the studies employed short-duration, high-intensity, low-frequency rTMS over M1 with variable duration and outcomes (Dafotakis et al., 2011; Garcin et al., 2013; Pollak et al., 2014). Consistently, low-frequency rTMS paradigm over M1 has been shown to improve MD symptoms in keeping with a decrease in intracortical inhibition of M1 (Siebner et al., 1999). However, the lack of blinded assessment and control groups and the non-homogeneity of stimulation setup (including TMS intensity and frequency, number of pulses, stimulation modality, and targeted area) make still unclear the therapeutic benefit and the neurophysiological underpinnings of rTMS in PMD management (Pollak et al., 2014). Furthermore, other promising cortical targets have been proposed, including the premotor cortex (PMC) owing to its widespread, bilateral, cortical−subcortical motor network subtending movement execution, even at a psychogenic level (Hallett, 2010, 2017; Huang et al., 2010; Voon et al., 2010; Mehta et al., 2013; Espay et al., 2018). However, this issue remains to be tested formally. In this regard, innovative approaches in non-invasive brain stimulation to manage patients with PMD are welcomed (including case reports, consistently with the objective difficulty to tailor randomized clinical trials). Herein, we report on a 50-year-old woman with a diagnosis of psychogenic myoclonus, who was managed with a long-duration, low-intensity (slightly suprathreshold), low-frequency (1 Hz) rTMS protocol over the left PMC, then assessing the clinical and EEG aftereffects. We found a significant reduction of involuntary movement frequency and intensity and of the related disability burden, which was paralleled by a large reshape of source current density within and connectivity between the frontal and parietal areas.

## Case Description

A 50-year-old female complained of a mild, progressive, bilateral weakness of legs, then involving arms, with mild distal paresthesia and pain at upper and lower limbs, and decreased deep tendon reflexes, which progressively developed over 1 month after the flu that occurred in early August 2019. There were no cranial nerve involvement or autonomic, bowel, or bladder dysfunctions. Personal and past clinical history was unremarkable. She was hospitalized in an acute neurological unit in mid-September 2019, where she was diagnosed with polyradiculoneuropathy. This was consistent with the clinical history, the electromyography data (evidence of proximal demyelination and axonal damage with denervation in both upper and lower limbs suggesting of a form of demyelinating Guillain-Barré syndrome-acute inflammatory demyelinating polyradiculoneuropathy form with secondary axonal loss), and the evidence of a mild enhancement of spinal nerve roots on gadolinium-enhanced axial T1-weighted images. She refused a cerebrospinal fluid examination. After the provision of general care treatment (as she refused intravenous immunoglobulin and plasma exchange, given the mild symptomatology), she was admitted to our rehabilitation unit to undergo intensive rehabilitation training. At the admission (early October 2019), she was able to get from lying to sitting, to weight-bear, and stand up only with aid, and to propel a wheelchair, whereas she was unable to stand alone and walk. Sensory disturbances were improved. Furthermore, she complained of sudden, involuntary muscle jerking of right thigh muscles when she was both resting and standing up. The patient was not taking any medication. Her Muscle Research Council (MRC; which grades muscle power on a scale of 0–5 in relation to the maximum expected for that muscle) was 26/60 (specifically, three muscles were tested in each extremity using the 0–5 MRC scale). Her Functional Independence Measure (FIM) score (an 18-item tool assessing physical, psychological, and social function to estimate the level of disability of a patient as well as a change in patient’s status in response to rehabilitation or medical intervention) was 68/126. The Hamilton Depression Rating Scale (HDRS =11) indicated a mild depression (Zigmond and Snaith, 1983). The Toronto Alexithymia Scale was within the normal range (<61; Demartini et al., 2014). Thigh myoclonus was assessed using the sum of the specific subscores of the Unified Myoclonus Rating Scale (UMRS; Frucht et al., 2002; 34/56). The UMRS has 73 items, grouped into five sections (patient’s questionnaire, frequency, and amplitude of myoclonus at rest and with action, stimulus sensitivity, functional tests, global disability scale, and presence and severity of negative myoclonus). This scale has satisfactory internal consistency, inter-rater reliability, and responsiveness to changes due to treatment when assessing the severity and characteristics of the disorder and the associated disability (Hainque et al., 2016).

She was trained with conventional physiotherapy (60-min), robot-aided gait training using the LokomatPro (Hocoma; Volketswil, Switzerland; 60-min), and walking and going upstairs/downstairs using the G-EO System (Reha Technology; Olten, Switzerland; 60-min), six times a week for 6 weeks. At the discharge (mid-November 2019), she was able to get from lying to sitting and to weight-bearing stand unaided, to stand up with aid, and to walk with two elbow crutches and supervision. The MRC was 34/60, the FIM 83/120. However, the involuntary movements of the right lower limb persisted unchanged and still significantly impaired the quality and the autonomy of gait, although the symptom was not associated with the clinical picture of the polyneuropathy. Conduction velocity studies showed mild signs of demyelination. A combination of findings suggested the psychogenic nature of the myoclonus, including the clinical features incongruous with organic myoclonus (inconsistent in frequency and amplitude, and involving more than one muscle groups), the movement improvement with distraction maneuvers, and the incongruous sensory loss or weakness (Fahn and Williams, 1988; Peckham and Hallett, 2009). Furthermore, the patient was provided with several electrophysiological examinations to assess organic myoclonus (Peckham and Hallett, 2009), which were all negative, including EEG (no potentials associable with epileptic myoclonus), TMS testing (normal cortical responsiveness to paired associative stimulation, normal intracortical facilitation and inhibition, cortical silent period (CSP) duration, and motor evoked potential (MEP) amplitude and recruitment curve), and surface EMG (showing long bursts of muscle activation with a highly variable agonist-antagonist muscle relationship).

The patient also underwent a resting-state EEG recording to assess brain connectivity parameters. We used a standard, digital 19-channel scalp EEG device, with the ground on the forehead and the reference on both the mastoids (Brain-Quick System; Micromed, Mogliano Veneto, Italy). EEG data were sampled at 512 Hz, filtered at 0.3–70 Hz (with 50 Hz notch). Electrode impedance was kept always below 5 kΩ. EEG recording lasted 10 min in a closed-eye state. EEG was cleaned from artifact offline by visual inspection and ICA-based rejection. The resulting artifact-free EEG was segmented in 2-s epochs (obtaining 255 epochs) and subjected to source current density analysis, which was conducted using the DIPFIT functions within EEGLAB (Oostenveld and Oostendorp, 2002). We constructed an equivalent current-dipole model explained the scalp topography of each IC using a boundary element head model based on the Montreal Neurological Institute (MNI)-152 template. The ICs were selected for further analyses whether the projection of the equivalent current dipole model to the scalp accounted for more than 80% of the scalp map variance. The artifactual nature of ICs was assessed consistently with the power spectra and the locations of their equivalent current dipoles (Jung et al., 2000a,b). The so-obtained electrocortical sources were subjected to functional connectivity analysis using the lagged phase synchronization (LPS), which is a method of nonlinear functional connectivity (Pascual-Marqui et al., 2011). Specifically, LPS estimates the similarity between signals in the frequency domain, based on normalized Fourier transforms. Thus, LPS represents the connectivity of two signals after excluding the instantaneous zero-lag component (i.e., a lot of artifact elements). This is necessary as both scalp and tomography (estimated intracranial) EEG signals can be biased by non-physiological, artifactual components (including volume conduction; Martins et al., 2016). The electrocortical sources’ density and connectivity were analyzed using a statistical nonparametric mapping method and a non-parametric permutation/randomization procedure (Holmes et al., 1996; Nichols and Holmes, 2002).

**Figure 1:**
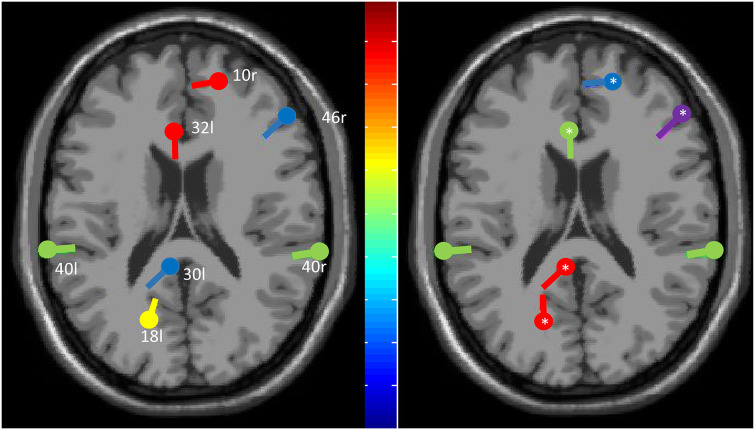
The plot of the equivalent dipoles (center) for the maximally independent brain source components (IC), i.e., those with a residual variance below 30% before and after Repetitive transcranial magnetic stimulation (rTMS) treatment (*means a statistically significant change, *p* < 0.001) across the 19-channel component scalp map based on fitting the measured 2D electrode locations to an individualized three-shell boundary element method head model. IC is color-ranked in order of variance contributed to the scalp data (colors show significant deviations in log power (dB) from baseline; green indicates no changes). Given that all the residual variances were low, the component maps are compatible with an origin in a single cortical patch.

**Figure 2:**
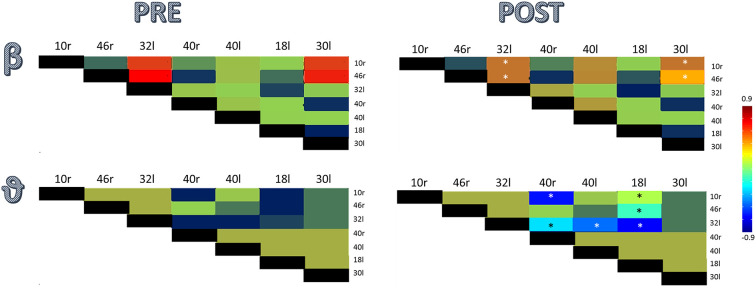
Component-wise correlation coefficients (among the significant ICs outlined in Figure 1), color-coded by whether ICs’ correlation coefficient is ranging from −0.9 to 0.9, before and after rTMS treatment (*means a statistically significant change, *p* < 0.001).

Data analysis illustrated an abnormal source current density within the middle frontal area (Brodmann area—BA-46), the ACC (BA32), the anterior-most portion of the prefrontal cortex (BA10), the Visual Association Area (BA18), the left and right supramarginal gyrus (BA40), and the agranular retrolimbic area (BA30; Figure 1). Furthermore, we found a beta hyperconnectivity between the prefrontal cortex and ACC, and a theta hypoconnectivity among limbic area, prefrontal cortex, and ACC (Figure 2).

After the diagnosis of psychogenic myoclonus was reached, the patient was invited to undergo rTMS, as she also refused to take any psychoactive drugs or undergo psychotherapy. Informed consent was obtained by the patient before initiating the experimental protocol. First, we measured the resting motor threshold (RMT) from the right rectus femoris muscle (rRF) by stimulating the medial leg area of the M1 contralateral to the affected limb. We then measured the MEP amplitude from the rRF at rest and during voluntary contraction to measure the CSP duration. Monophasic TMS pulses were given through a standard 90 mm figure-of-eight shaped coils connected to a high-power Magstim 200 stimulator (Magstim Company Limited; Whitland, Dyfed, UK) for these measurements. A 1 Hz rTMS protocol was delivered through a standard 90 mm figure-of-eight shaped coils wired to a Magstim Rapid stimulator (Magstim Company Limited; Whitland, Dyfed, UK). The stimulation intensity was adjusted to 115% of RMT from rRF. We delivered 1,200 biphasic magnetic pulses in a single session at 1 Hz over a point sited at 2 cm anterior and 1 cm medial to the hotspot for MEP elicitation from rRF (Fink et al., 1997; Schluter et al., 1998; Murase et al., 2005; Borich et al., 2009). These parameters were chosen to inhibit the motor cortex (Bäumer et al., 2003; Valero-Cabre et al., 2008; Rossini et al., 2015). The patient underwent the rTMS treatment once a day, in the morning (approximatively between 9 am and 11 am), five times a week (from Monday to Friday) for 6 weeks. A fixation unit with an integrated head holder was built, upon which a flexible coil holder was mounted, to ensure constant stimulation conditions across sessions. The optimal coil position was drawn on the scalp, and the constancy of coil positioning was continuously monitored throughout the sessions. TMS setup and coil positioning were checked before starting and during every TMS session.

At the end of December 2019, the patient was able to stand up from an average height chair, to walk safely indoors with one elbow crutch, and to ascend and descend a flight of stairs with a rail. The magnitude and frequency of the myoclonus were strongly reduced as per the UMRS score (14/56). The MRC was 35/60, the FIM was 98/120. Last, all source current densities were modified by the rTMS treatment but left and right BA40. Specifically, we found a source magnitude decrease in BA10R, BA32L, and BA46R (all *p* < 0.001) and an increase in BA30L and BA18L (both *p* < 0.001; Figure 1). In parallel, the rTMS treatment reset the beta hyperconnectivity between the prefrontal cortex and ACC and the theta hypoconnectivity between limbic area, prefrontal cortex, and ACC (all *p* < 0.001; Figure 2). After 2 months (February, 2020), the patient’s clinical conditions were unvaried.

## Discussion

To the best of our knowledge, this is the first time that the PMC contralateral to the affected side was triggered by a low-frequency rTMS in PMD, including psychogenic myoclonus. Most of the rTMS studies in patients with PMD employed high intensity, low-frequency pulses over either the M1 contralateral to the affected side of the vertex, with variable outcomes and duration of the aftereffects. Further, only a few neurophysiological data are reported in the literature concerning rTMS aftereffects in PMDs (Pollak et al., 2014).

One could have concerns about the correctness of the diagnosis. Indeed, the diagnosis of psychogenic myoclonus was based on the clinical and DSM-V criteria (including the inconsistency/incongruency, abrupt onset, distractibility, and oddness of involuntary movements; Fahn and Williams, 1988; Williams et al., 1995; Kranick et al., 2011; American Psychiatric Association, 2013). Furthermore, myoclonus dramatically reduced after distraction maneuvers and temporarily disappeared after suggestion maneuvers (Fahn and Williams, 1988; Williams et al., 1995). Last, we recorded some neurophysiological data, including surface EMG that documented the inconsistency of frequency and amplitude of muscle jerks involving more muscles, being characterized by burst length >70 ms, and showing a triphasic pattern on agonist/antagonist muscles (Brown and Thompson, 2001; Monday and Jankovic, 1993). Unfortunately, we did not have the opportunity to ascertain the presence of a Bereitschafts potential. However, a Bereitschafts potential by itself is not a univocal method to determine whether myoclonus is a psychogenic (Hallett, 2010).

The patient reported an improvement in myoclonus severity already after 2 weeks of rTMS treatment. This improvement consolidated at the end of the rTMS paradigm and persisted up to 2 months after the discharge. Indeed, the rehabilitation treatment significantly improved the motor outcome, whereas the rTMS protocol specifically improved the PMD and had partial effects on the motor outcome as compared to the previous rehabilitation treatment (Czarnecki et al., 2012; McCormack et al., 2014; Nielsen et al., 2015, 2016). Noteworthy, the patient did not report any side effects during or after the end of the rTMS treatment.

The exact mechanism of action of rTMS on PMD is still unclear. The available studies suggest that rTMS may work by inducing non-specific cognitive-behavioral rather than neuromodulator effects (Garcin et al., 2017), although they used different TMS setups with regard to coil shape, placebo stimulation, stimulation duration, and the number of sessions and stimuli. In particular, a small number of pulses with high stimulation intensity has been usually adopted in such studies to cause muscle twitching. Therefore, it seems unlikely that such an rTMS can result in a long-term plastic effect. Such a stimulation modality may raise complex placebo effects caused by the external triggering of movement (Garcin et al., 2013; Pollak et al., 2014; Ricciardi and Edwards, 2014). Conversely, the long-lasting effects of the slightly suprathreshold rTMS we achieved on psychogenic myoclonus may suggest modulation of corticospinal excitability in keeping with a long-term depression-like synaptic plasticity mechanism (Bäumer et al., 2003; Valero-Cabre et al., 2008; Rossini et al., 2015). Indeed, at baseline, the patient showed a low activity of the posterior areas, a frontal hyperactivation, a beta hyperconnectivity between the prefrontal cortex and ACC, and a theta hypoconnectivity between limbic area, prefrontal cortex, and ACC. This scenario suggests a detrimental drive from the motor association areas and prefrontal cortex to the primary motor cortex and the concomitant failure of a feed-forward mechanism, which all account for psychogenic movement generation (Hallett, 2010, 2017; Voon et al., 2010; Mehta et al., 2013; Espay et al., 2018; Baizabal-Carvallo et al., 2019). These abnormalities were reset partially by rTMS treatment on the PMC. It is hypothesizable that rTMS interfered with the abnormal prefrontal cortical activation (beta hyperconnectivity) due to the lack of a posterior-to anterior brain areas modulation (theta connectivity failure). The lack of a posterior-to anterior modulation may be the basis on which the PMC is disconnected from the rest of the motor network, thus driving aberrant movements (Baizabal-Carvallo et al., 2019). A hypoactivity of the SMA over the temporoparietal junction, which normally compares what has been planned and what has been done by a motoric point of view, may also contribute to this premotor-motor impairment. Furthermore, a contribution by a disconnected sensitive-emotional network (including striatum and amygdala) from the motor network could also be hypothesizable (Edwards et al., 2012; Schrag et al., 2013; Baizabal-Carvallo et al., 2019). Therefore, rTMS-induced PMD improvement may also depend on a real neuromodulation effect, rather than a cognitive-behavioral effect alone. Overall, it is hypothesizable that the rTMS approach improved PMD by combining cortical neuromodulation (including changes in local connectivity between the brain areas directly, including PMC and ACC, and indirectly involved, including limbic and visuomotor areas, in PMD expression) and non-specific mechanisms suggesting cognitive-behavioral effects (e.g., environment and rehabilitation personnel).

The present case has some limitations. First, the lack of a sham control treatment, the short follow-up period, and the short interval between the first and second phases of treatment limits the power of the conclusions, since a placebo effect cannot be excluded, and prevent us from estimating the duration of the aftereffects induced by each intervention alone. Thus, the claimed long-term benefits deserve further controlled study, regardless of the mechanisms of rTMS aftereffects. However, we carefully kept the patient unaware whether the rTMS was introduced as purely diagnostic, therapeutic, or both (owing to the expectancy that is generated by the therapeutic encounter; Stone and Edwards, 2011). Second, the lack of extensive neurophysiological measures but EEG, so that the exact mechanism of action of rTMS on PMD needs to be clarified further. However, our case was aimed at preliminarily assessing the safety and effectiveness of this intervention and its ability to prevent any relapse of PMD (even though in a short period). Last, we were not able to submit the patient to a video-EEG recording, which could have been significant toward the differential diagnosis of psychogenic and non-psychogenic disturbance. However, we believe that the extensive clinical and neurophysiological assessment we conducted confirms the psychogenic nature of the movement disorder in our patient.

In conclusion, following the promising results of this case-report, it seems reasonable to verify the effectiveness and the mechanism of action of low-frequency rTMS over PMC in larger samples of patients with PMD, as it seems a safe and valuable approach for the management of such complex and rather common disorders.

## Data Availability Statement

The raw data supporting the conclusions of this article will be made available by the authors, without undue reservation.

## Ethics Statement

The studies involving human participants were reviewed and approved by the Institutional Review Board of IRCCS Centro Neurolesi Bonin Pulejo. Written informed consent was obtained from the participants for the publication of this case report, including any identifiable information included in the study.

## Author Contributions

LP, LB, BP, and SP: substantial contributions to the conception or design of the work; or the acquisition, analysis or interpretation of data for the work. AN, PT, and RC: drafting the work or revising it critically for important intellectual content. LP, LB, BP, SP, AN, PT, and RC: provide approval for publication of the content. RC and PT: agree to be accountable for all aspects of the work in ensuring that questions related to the accuracy or integrity of any part of the work are appropriately investigated and resolved.

## Conflict of Interest

The authors declare that the research was conducted in the absence of any commercial or financial relationships that could be construed as a potential conflict of interest.
